# CRISPR/Cas-Based Nanobiosensor Using Plasmonic Nanomaterials to Detect Disease Biomarkers

**DOI:** 10.1007/s13206-024-00183-x

**Published:** 2025-02-13

**Authors:** Jin-Ha Choi, Jinho Yoon, Meizi Chen, Minkyu Shin, Li Ling Goldston, Ki-Bum Lee, Jeong-Woo Choi

**Affiliations:** 1 School of Chemical Engineering, Jeonbuk National University, 567 Baekje-daero, Deokjin-gu, Jeonju-si, Jeollabuk-do 54896 Republic of Korea; 2 Department of Biomedical-Chemical Engineering, The Catholic University of Korea, 43 Jibong-ro, Wonmi-gu, Bucheon-Si, Gyeonggi-Do 14662 Republic of Korea; 3 Department of Chemistry and Chemical Biology, Rutgers, The State University of New Jersey, 123 Bevier Road, Piscataway, NJ 08854 USA; 4 Department of Chemical & Biomolecular Engineering, Sogang University, 35 Baekbeom-ro, Mapo-gu, Seoul, 04107 Republic of Korea

**Keywords:** CRISPR/Cas system, CRISPR/Cas-based nanobiosensors, Nanotechnology-enabled biosensing, Plasmonic nanomaterials, Collateral effect

## Abstract

The development of clustered regularly interspaced short palindromic repeats (CRISPR) and CRISPR-associated protein (Cas) technology (CRISPR/Cas) as a gene-editing tool has the potential to revolutionize nucleic acid analysis. Recently, CRISPR/Cas systems have demonstrated considerable promise in the development of biosensors for the detection of essential disease biomarkers because they exhibit nonspecific collateral cleavage properties upon target sequence recognition. However, the CRISPR/Cas-based biosensors developed thus far have limitations, such as complicated steps, low sensitivity, low selectivity, and low signal-to-noise ratios. These limitations can be overcome by incorporating the unique characteristics of plasmonic nanomaterials into CRISPR/Cas systems to enhance the signal and improve the sensitivity of these biosensors. From this perspective, current interdisciplinary studies on CRISPR/Cas-based nanobiosensors comprising plasmonic nanomaterials can contribute to the development of highly sensitive CRISPR/Cas-based nanobiosensors. These nanobiosensors can detect attractive disease biomarkers, such as viral nucleic acids, small molecules, and proteins. This review article provides a thorough overview of nanobiosensors that incorporate CRISPR/Cas systems combined with plasmonic nanomaterials to enhance biosensing performance. We believe this review will inspire novel approaches and further innovation in the fields of molecular diagnostics and biomedicine aimed at using CRISPR/Cas systems and plasmonic nanomaterials for more personalized and effective medical treatments.

## Introduction

Among the various gene-editing strategies, clustered regularly interspaced short palindromic repeats (CRISPR)-based gene editing stands out as a versatile approach that is used across various applications. It encompasses controlling transcription, modifying epigenomes, conducting genome-wide screens, and even enabling chromosome imaging [1–3]. This gene-editing method has sparked substantial interest in both the scientific and medical communities because of its potential therapeutic applications, which include the treatment of genetic disorders and cancers [4]. These CRISPR/Cas systems have been used to facilitate genome and transcriptome editing by inducing RNA cleavage at specific user-defined locations or double-stranded DNA (dsDNA) breaks within living cells [5]. Adapting these systems to conduct gene editing at various genomic loci requires altering the sequence of the single guide RNA (sgRNA) within a CRISPR RNA (crRNA). CRISPR/CRISPR-associated protein (Cas) systems use crRNAs to guide Cas enzymes in recognizing and cleaving nucleic acid targets during gene editing. The crRNA can form a hybrid with precise regions of interest within the DNA or RNA through complementary base pairing. In certain systems, this binding is limited to the vicinity of an adjacent protospacer motif or a sequence flanking the protospacer [6]. CRISPR/Cas systems are increasingly being used for therapeutic purposes as biomedical field of research expands. To date, CRISPR/Cas systems have been employed in therapeutic applications, such as targeted genome downregulation and protein inhibition [7–9].

CRISPR/Cas systems have been found to offer immense potential in the field of biosensors because they exhibit nonspecific collateral cleavage properties upon target sequence recognition, which is one of their inherent characteristics. In details, bacteria use CRISPR arrays to defend against bacteriophages by storing small pieces of bacteriophage DNA in their genome as a record of past infections. When the bacteriophage attacks again, the bacteria use the Cas proteins to identify and cut the bacteriophage DNA preventing infection. This natural defense mechanism was adapted by scientists to create CRISPR-Cas9, a tool that can precisely cut and edit DNA in other organisms. The discovery has revolutionized genetics, allowing for targeted genetic modifications in research, medicine, and agriculture. In particular, these systems can be employed in a variety of biomedical fields and have recently gained widespread attention because of their potential for use in the development of biosensors [10]. Notably, the field of CRISPR/Cas system-based diagnostics has made improvements to the programmability, specificity, and usability of the CRISPR technique. Its goal is to develop nucleic acid-based point-of-care testing for regular clinical use in order to eliminate the need for polymerase chain reaction (PCR) [11]. Nucleic acid-based testing has emerged as the benchmark for highly sensitive detection of diseases, particularly viral infections. Moreover, quantitative PCR has gained popularity for its ability to accurately identify a small number of DNA or RNA molecules, thereby definitively diagnosing specific diseases. Nevertheless, this method is challenging to broadly implement in both clinical and commercial environments owing to its complexities. Conversely, CRISPR/Cas-based biosensors have distinct advantages, including collateral cleavage property, rapid response, enhanced integrity, and simplicity [9, 12]. Additionally, they can be used for non-nucleic acid target detection [13]. The development of CRISPR/Cas-based biosensors has been groundbreaking; however, the inherent limitations of low biomolecule concentrations in nature, such as weak signal generation and limited stability, have led to reduced sensitivity, selectivity, and signal-to-noise ratios, particularly in the absence of nucleic acid amplification steps.

**Fig. 1:**
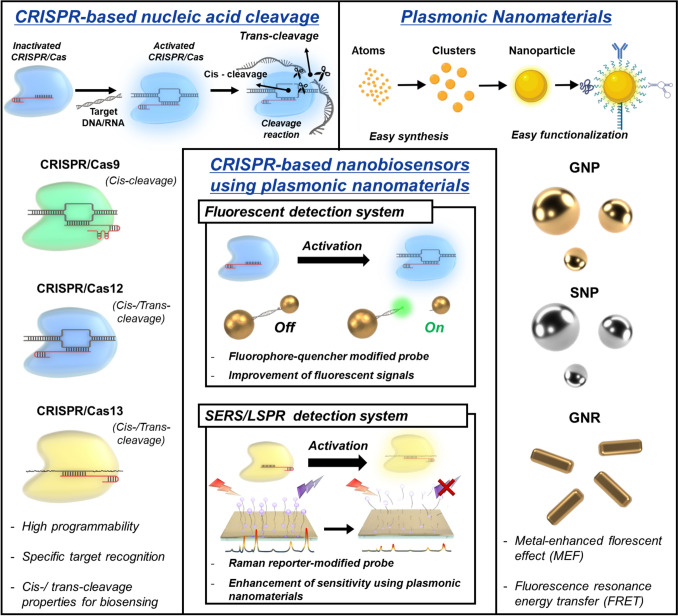
CRISPR/Cas-based nanobiosensors that use plasmonic nanomaterials to detect disease biomarkers

One potential solution to the inherent limitations of CRISPR/Cas systems for biosensing is to incorporate plasmonic nanomaterials into these systems. For example, integrating CRISPR/Cas systems with plasmonic nanomaterials can enhance signal amplification and increase the sensitivity of CRISPR/Cas-based biosensors. This is enabled by the inherent properties of plasmonic nanomaterials, such as light scattering and absorbance. These properties allow plasmonic nanomaterials to leverage the metal-enhanced fluorescence effect (MEF), which can improve sensitivity [14, 15]. CRISPR/Cas-based nanobiosensors composed of plasmonic nanomaterials are broadly utilized in the field of biosensors and can diagnose attractive disease biomarkers, including viral nucleic acids, small molecules, and proteins [16–20]. From this perspective, a selective overview of recent relevant studies is required to highlight the properties of plasmonic nanomaterials and their usability for the development of CRISPR/Cas-based nanobiosensors. The provision of such information can suggest a novel approach and offer an in-depth interdisciplinary knowledge of CRISPR/Cas systems comprising plasmonic nanomaterials, which has not been offered in other review papers on CRISPR/Cas-based biosensors that have been published thus far. This review provides interdisciplinary knowledge of CRISPR/Cas-based nanobiosensors composed of plasmonic nanomaterials. It covers the biosensing mechanisms of CRISPR/Cas systems, widely used plasmonic nanomaterials, and CRISPR/Cas integrated with plasmonic nanomaterials, and recently reported CRISPR/Cas-based nanobiosensors for the detection of disease biomarkers (Fig. 1).

## CRISPR/Cas Systems for Biosensing Applications

**Fig. 2:**
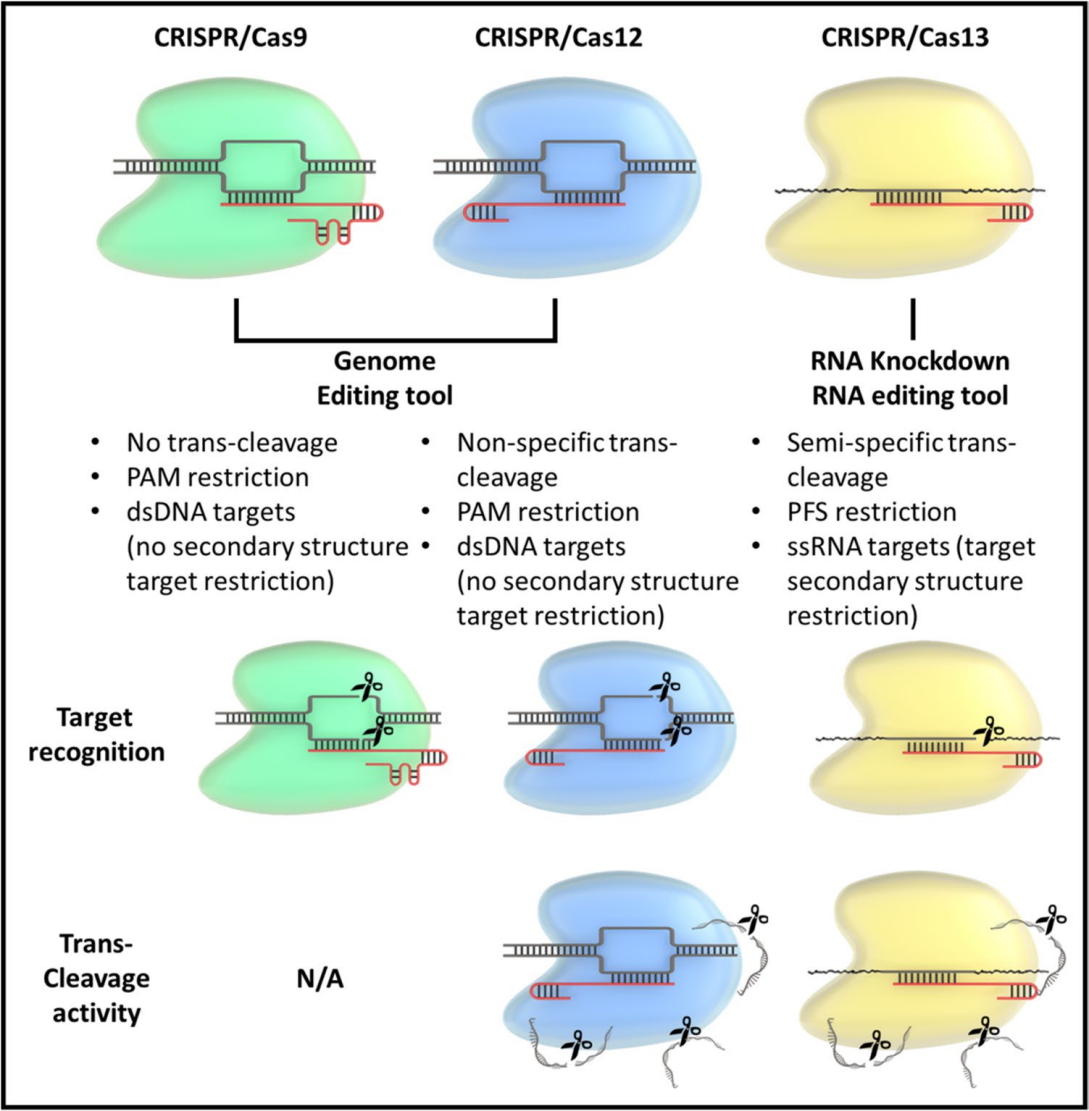
Representative mechanisms of CRISPR/Cas-based nucleic acid detection. Target nucleic acids are degraded by Cas enzymes activated after complementary binding to the crRNA (cis-cleavage). Additionally, the RNP complex bound to the target nucleic acid randomly decomposes surrounding ssDNA or ssRNA (trans-cleavage)

Several CRISPR/Cas systems can be distinguished by their enzymatic properties (Fig. 2 and Table 1). The primary difference between CRISPR type II (Cas9) systems, CRISPR type V (Cas12) systems, and CRISPR type VI (Cas13) systems is that the latter two systems can induce nonspecific collateral cleavage (trans-cleavage) after target identification [21–23].

**Table 1:** First-generation CRISPR/Cas-based biosensors

Technology name	Cas type	Target	Signal amplification	Detection method	Sensitivity	Reference
Cas9 triggered exponential amplification reaction (CAS-EXPAR)	Cas9	DNA/RNA	Exponential amplification reaction (EXPAR)	Fluorescence	aM	[30]
DNA endonuclease targeted CRISPR trans reporter (DETECTR)	Cas12a	DNA	Recombinase polymerase amplification (RPA)	Fluorescence	aM	[26]
One-hour low-cost multipurpose highly efficient system (HOLMES)	Cas12a	DNA/RNA	Polymerase chain reaction (PCR); Reverse transcription-RPA	Fluorescence	aM	[28]
HOLMESV2	Cas12b	DNA/RNA	Loop-mediated isothermal amplification (LAMP); Reverse transcription-LAMP; asymmetric PCR	Fluorescence	aM	[29]
Specific high sensitivity enzymatic reporter unlocking (SHERLOCK)	Cas13a	DNA/RNA	RPA	Fluorescence	aM	[20]
SHERLOCKV2	Cas13a; Cas12a	DNA/RNA	RPA	Fluorescence; colorimetric	zM	[31]

CRISPR/Cas complexes are widely known for their ability to recognize and cleave target DNA or RNA strands through a process known as cis-cleavage, which is activated by an enzymatic effect. Moreover, this collateral activity involves the degradation of randomized single-stranded DNA (ssDNA) or single-stranded RNA (ssRNA) within a mixture. In this process, nucleic acids can be detected through signal amplification. Additionally, an array of readout techniques can be employed by introducing functionalized reporter nucleic acids that are cleaved as a result of the collateral activity [24, 25]. The CRISPR/Cas13 enzyme family has a size range of approximately 900–1300 amino acids. These enzymes are adept at detecting ssRNA in a cis-conformation, and they exhibit cis-cleavage activity toward the target RNA. Additionally, they demonstrate collateral trans-cleavage activity against ssRNA in vitro [20]. In the Cas13-based assay known as specific high-sensitivity enzymatic reporter unlocking (SHERLOCK), the DNA or RNA undergoes isothermal amplification using recombinase polymerase amplification (RPA) or reverse transcription-RPA (RT-RPA). A promoter facilitates the RNA transcription of the target, which is subsequently recognized and bound by a complex of Cas13a and crRNA that bears a complementary sequence to the target. Upon activation, Cas13 degrades both the target RNA through cis-cleavage and the nontarget ssRNA probe via collateral trans-cleavage. These ssRNA probe molecules consist of a fluorophore and a quencher linked by a short RNA oligomer. The cleavage of the probe causes the fluorophore and quencher to separate, resulting in fluorescence emission. These series of reactions enable the detection of bacterial DNA, viral RNA, cancer-associated mutations, and single-nucleotide polymorphisms with exceptional attomolar (10^−18^ M) sensitivity.

Similarly, Cas12 enzymes have been used as a CRISPR-based diagnostic system to detect target dsDNA and ssDNA [26, 27]. The detection strategy involving Cas12a, which is derived from the Lachnospiraceae bacterium (LbCas12a) or other microbes, is very similar to that involving Cas13a discussed above. One of the first Cas12a-based methods is DNA endonuclease-targeted CRISPR transreporter (DETECTR), which measures the complementary binding of the target dsDNA to the crRNA of the ribonucleoprotein (RNP) complex. When Cas12a cleaves the target dsDNA (cis-cleavage), it also randomly cleaves the surrounding ssDNA (trans-cleavage), resulting in a collateral effect. Fluorescence emission is generated when Cas12a degrades ssDNA probes with a fluorophore and quencher attached to both ends. The target DNA can be measured quantitatively using the fluorescence intensity. The DETECTR method uses RPA to amplify the target DNA, enabling highly sensitive measurements. Conversely, the one-hour low-cost, multipurpose highly efficient system (HOLMESv2) uses Cas12b derived from *Alicyclobacillus acidoterrestris* (AacCas12b) as well as loop-mediated isothermal amplification (LAMP) instead of RPA to amplify the target DNA and provide measurement sensitivity as low as 10 aM [28, 29]. Both DETECTR and HOLMESv2 exhibit excellent attomolar sensitivity for detecting target DNAs. However, most CRISPR/Cas-based biosensing systems, including DETECTR and HOLMES, require preamplification to achieve this level of sensitivity for early diagnosis. This preamplification step increases system complexity and presents challenges for the widespread adoption of these types of systems. As these two systems demonstrate, an essential requirement for establishing a functional sensing system is the use of two distinct enzymes: a polymerase for target amplification and a Cas enzyme for target recognition. It is also crucial to optimize the specific conditions and reaction steps for each enzyme to achieve the best outcome.

## Plasmonic Nanomaterials for Biosensing

Plasmonic nanomaterials have been widely used to develop highly sensitive biosensors for biomarker detection because of their distinct characteristics [32–34]. Surface plasmon resonance (SPR) is a phenomenon in which electromagnetic waves resonate with the collective oscillations of unbound electrons on the surfaces of plasmonic materials, such as gold and silver [35, 36]. This resonance occurs at the boundary between dielectric and plasmonic substances, resulting in intensified and concentrated incident light. Owing to these attributes, plasmonic nanomaterials are remarkably sensitive to changes in surface dielectric properties, making them suitable for use in advanced diagnostic platforms. Among the various plasmonic nanomaterials, Au and Ag have been extensively researched for use in molecular diagnostic platforms. The interplay between resonant incident light and collective electron oscillations on the surface of these noble nanomaterials yields amplified or modified optical absorption and pronounced scattering effects, particularly when their particle sizes exceed a few tens of nanometers. The dimensions, configuration, and composition of these plasmonic nanomaterials significantly influence the degree of enhancement, modification, and scattering efficiency.

In biosensing applications, the interplay between biomolecules and plasmonic materials triggers alterations in various optical attributes, such as the plasmonic resonance wavelength and absorbance. By precisely and sensitively gauging these modified optical properties, it is possible to accurately detect biological materials, including biomarkers. The interaction between plasmonic nanoparticles (NPs) and biomolecules is particularly notable because it produces four distinct effects: first, the inherent emission intensity of a fluorescent material diminishes (quenching effect) when in close proximity to plasmonic NPs. Second, fluorescence resonance energy transfer (FRET) causes shifts in the wavelength of emitted light owing to energy exchange between the fluorescent molecule and the plasmonic nanomaterial. Third, the emission intensity of a fluorescent molecule increases when plasmonic nanomaterials are present [37]. Plasmonic nanostructures offer the added benefit of increasing the electromagnetic fields in relation to the incoming light. The confinement of the nanostructure influences the level of enhancement. Nanostructures with sharp edges and vertices create localized field enhancements known as “hot spots.” These hot spots play a critical role in surface-enhanced Raman scattering (SERS), a technique that enables the highly sensitive detection of molecular vibrational modes [38]. Researchers have made several attempts to leverage these plasmonic properties to enhance the detection of biomarkers, such as viral nucleic acids, for the early detection of fetal diseases and other medical conditions. The following sections will discuss recently developed CRISPR/Cas-based plasmonic biosensors, with a focus on CRISPR/Cas-based biosensing applications.

## CRISPR/Cas-Based Fluorescent/Colorimetric Nanobiosensors for Detecting Disease Biomarkers

**Fig. 3:**
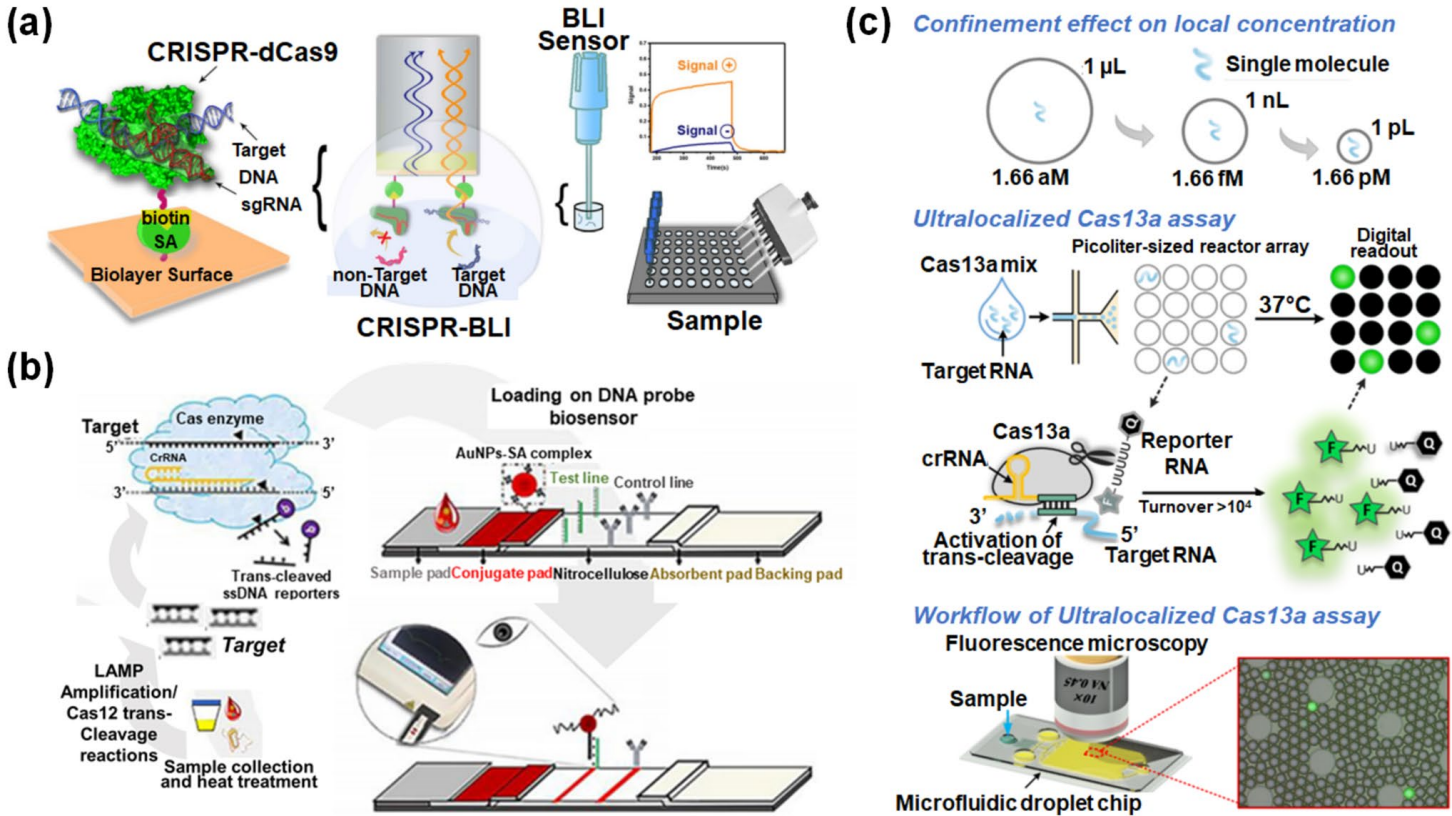
CRISPR/Cas-based fluorescent/colorimetric nanobiosensors. **a** Schematic of the CRISPR/dCas9-mediated interferometry biosensing platform for detecting target nucleic acids through changes in the thickness of an optical layer. Reproduced with permission [43]. Copyright 2021, John Wiley and Sons. **b** Schematic of the CRISPR/Cas12a-based LFA biosensor for detecting HPV16 and HPV18 DNA through LAMP. Reproduced with permission [48]. Copyright 2020, Elsevier. **c** Schematic of the confinement effect for promoting the local molecular concentration of samples, the fabrication of picoliter-sized droplet reactors, and the fluorescent biosensing process for miRNA-17 and 16S rRNA detection. Reproduced with permission [61]. Copyright 2021, American Chemical Society

Numerous studies have been conducted to develop fluorescent or colorimetric biosensors using CRISPR/Cas systems in order to detect disease biomarkers, such as nucleic acids and proteins [17–19, 39–42]. In particular, many studies have focused on the regulation of the emission intensity of fluorescent molecules through the cleavage by activated CRISPR/Cas after target detection. For example, some researchers developed a CRISPR/dCas9-mediated interferometry platform that detects nucleic acids by measuring the change in the thickness of its optical layer upon target detection (Fig. 3a) [43]. The CRISPR/dCas9 complex was immobilized on a substrate via a biotin–streptavidin reaction, and it scanned and recognized a specific target sequence using sgRNA. After hybridization with the target sequence, the CRISPR/dCas9 biolayer on the substrate exhibited a change in its optical thickness. This resulted in a shift in the interference spectrum and a change in the optical output signal by biolayer interferometry. The developed biosensor exhibited rapid, accurate, and facile biosensing properties for nucleic acid detection with high sensitivity (limit of detection (LOD): 863.4 pM). Most CRISPR/Cas-based optical biosensors have been designed as turn-on biosensors that display fluorescent signals of dyes from the quenched state when the activated CRISPR/Cas complex cleaves the reporter nucleic acid after detecting the target [40, 44–48]. In most CRISPR/Cas-based optical biosensing applications, the reporter molecule is an ssDNA reporter modified with a fluorescent dye and a quenching material on either end. The generated fluorescent signal enables optical biosensing. Once the CRISPR/Cas12a enzyme is activated by the target DNA, it cleaves the ssDNA. Consequently, the fluorescent dye is released from the quenching material, which displays fluorescent signals and confirms the presence of the target [49, 50]. In one study, the authors developed a Cas12a-based visual detection biosensor (Cas12aVDet) by introducing quenched fluorescent ssDNA reporters (FAM-TTATT-BHQ1). The CRISPR/Cas12a-based fluorescent biosensor was successfully used to detect nucleic acids in mycoplasma-contaminated cell culture. The biosensor was integrated with RNA polymerase I to amplify the signal generated from the ssDNA reporter cleaved by activated Cas12a after nucleic acid detection. The fluorescent signal was visualized within 30 min [50]. In addition to fluorescent-type biosensors, lateral flow assay (LFA)-type biosensors have been studied using CRISPR/Cas systems. These LFA-type biosensors can provide simple and quick detection in any setting [51–55]. In one study, a CRISPR/Cas12a-based LFA biosensor was developed to detect viruses, particularly human papillomavirus (HPV)16 and HPV18, based on LAMP (Fig. 3b) [48]. After the LAMP process, the CRISPR/Cas12a was activated for the trans-cleavage of a biotin-modified ssDNA reporter in the presence of amplified HPV16 or HPV18 viral DNA, and the trans-cleaved ssDNA reporter could not immobilize on the complementary DNA-immobilized LFA. Contrastingly, in the absence of the target viral DNA, the non-trans-cleaved ssDNA reporter was immobilized on the LFA via DNA hybridization, and streptavidin-conjugated AuNPs were immobilized on the biotin-modified ssDNA reporter on the LFA. Thus, owing to the formation of the AuNP band on the test line, the target viral DNA could be detected at an attomolar LOD and be observed in real time with the naked eye. Moreover, target amplification strategies, such as tyramide signal amplification and DNA walker-mediated amplification, have been integrated with CRISPR/Cas12a to develop ultrasensitive optical biosensors [56, 57]. In addition to the CRISPR/Cas12a-based biosensors discussed here, a combination of CRISPR/Cas12a with functional nanomaterials has been developed in several recent studies. For example, MnO_2_ nanoflowers were incorporated with CRISPR/Cas12a to provide intracellular monitoring through miRNA detection [58]. Furthermore, a colorimetric CRISPR/Cas12a-based biosensor for detecting African swine fever virus (ASFV) was developed by simultaneously integrating AuNPs and magnetic beads with CRISPR/Cas12a [59]. Additionally, metal ions (Mn^2+^) were used to develop a Mn^2+^-activated CRISPR/Cas12a system that amplifies fluorescent signals in order to detect carbaryl insecticide [60].

One major issue with CRISPR/Cas13-based optical biosensors is their sensitivity. There are multiple approaches to increasing the sensitivity of CRISPR/Cas13-based optical biosensors. In a recent study, a novel droplet microfluidic system-mediated CRISPR/Cas13 fluorescent biosensor was designed to detect different types of RNAs, such as miRNA-17 and 16S rRNA [61]. This innovative method employs picoliter-sized droplet reactors, which help to enhance the local concentrations of the target, reporter, and CRISPR/Cas13. This enables high sensitivity without relying on traditional amplification processes. It also enables sensitive detection of the target analyte, considering that the overall sample volume is lowered when measuring the same target quantity. As shown in Fig. 3c, the CRISPR/Cas13-based biosensing assay in picoliter-sized droplets was designed to promote the local molecular concentration of samples in order to achieve a highly efficient reaction and detection. This biosensor exhibits a 10,000-fold higher sensitivity for single molecular level RNA quantification without amplification when compared to bulk-scale CRISPR/Cas13-based biosensing assays. Furthermore, a CRISPR/Cas13-triggered graphene oxide-based fluorescent biosensor has been recently developed [62]. Additionally, in place of functional nanomaterials, several novel DNA nanostructures, such as DNA walkers and DNA hydrogels, have been incorporated with CRISPR/Cas systems to develop highly sensitive biosensors [63–66]. From a broad perspective, various CRISPR/Cas-based fluorescent/colorimetric biosensors with improved target diversity and sensitivity have been developed [67]. These CRISPR/Cas-based optical biosensors are expected to exhibit enhanced performance when combined with plasmonic nanomaterials.

**Fig. 4:**
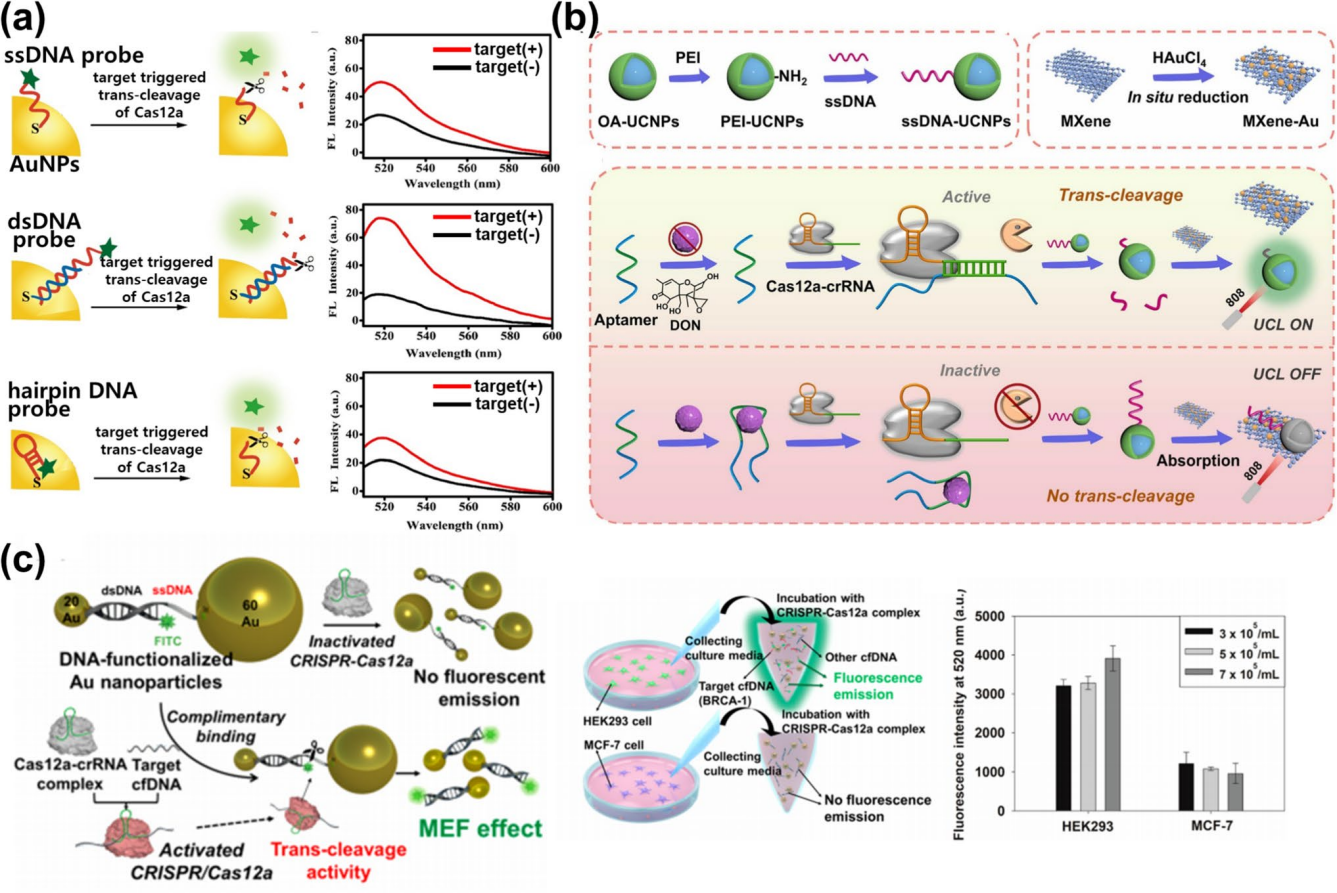
CRISPR/Cas-based fluorescent/colorimetric nanobiosensors using plasmonic nanomaterials. **a** Schematic and fluorescent intensities of the CRISPR/Cas12a-based fluorescent nanobiosensors based on AuNPs with fluorescent probes immobilized via ssDNA, dsDNA with a single-stranded part, and hairpin DNA. Reproduced with permission [68]. Copyright 2021, Elsevier. **b** Schematic illustration of the CRISPR/Cas-based fluorescent nanobiosensor composed of an MXene–Au hybrid and UCNPs for DON detection. Reproduced with permission [69]. Copyright 2022, Elsevier. **c** Schematic of the fluorescent biosensor based on the quenching and MEF effects of AuNPs for BRCA-1 gene detection. The BRCA-1 gene is detected after release from breast cancer cells. Reproduced with permission [70]. Copyright 2021, American Chemical Society

A more sensitive CRISPR/Cas-based nanobiosensor can be developed by integrating CRISPR/Cas systems and plasmonic nanomaterials, which can produce fluorescent or optical signals during target detection. Several studies have reported introducing plasmonic nanomaterials into CRISPR/Cas systems to develop biosensors. For example, a CRISPR/Cas12a-based fluorescent nanobiosensor was developed for the detection of nucleic acids using the plasmonic property of AuNPs capable of quenching the fluorescent signals of adjacent fluorophores [68]. The fluorescent probe was composed of a fluorescent dye (FAM) and immobilized on the AuNP via ssDNA, dsDNA with a single-stranded part, and hairpin DNA (Fig. 4a). Prior to cleavage by the activated CRISPR/Cas, the fluorescent signal was blocked by the quenching effect of the AuNP. However, in the presence of the target DNA sequence, the CRISPR/Cas was activated through hybridization with the target DNA, and then the activated CRISPR/Cas cleaved the ssDNA of the fluorescent probe. The FAM was released from the AuNP in every prepared condition, as the single-stranded parts were fully cleaved; this caused the FRET effect of the AuNP to disappear and allowed the fluorescent signal to be recovered. In another study, Au was decorated on the surface of MXene (Ti_3_C_2_T_x_), and the FRET property of this hybrid material was leveraged to develop a CRISPR/Cas-based fluorescent nanobiosensor for the detection of a non-nucleic acid target (deoxynivalenol (DON)) (Fig. 4b) [69]. The DON aptamer was introduced to detect the non-nucleic acid target using CRISPR/Cas. When DON was absent, the DON aptamer activated the CRISPR/Cas, which then cleaved the ssDNA on the surface of upconversion NPs (UCNPs). Thereafter, the fluorescent signals of the UCNPs were recovered and amplified by the MXene–Au hybrid. However, when DON was present, the DON aptamer combined with it and was therefore unable to activate the CRISPR/Cas. Next, the remaining ssDNA on the UCNP surface could induce the attachment on the MXene–Au hybrid. The fluorescent signals of the UCNPs were blocked because of the quenching by the MXene–Au hybrid. By utilizing the excellent FRET property of the MXene–Au hybrid, the developed nanobiosensor exhibited a sufficient LOD of 0.64 ng/mL for non-nucleic acid target detection.

In addition to the quenching effect derived from AuNPs, the MEF effect derived from plasmonic AuNPs has been exploited to develop a CRISPR/Cas-based fluorescent nanobiosensor for the detection of breast cancer gene-1 (BRCA-1) [70]. Different sizes of AuNPs were inserted and connected through partially dsDNA to generate both effects, and a fluorescent molecule (Fluorescein; FITC) was functionalized at the end of the ssDNA (Fig. 4c). Thus, in the state where the AuNPs were connected to each other through DNA, the fluorescent signal was quenched by the large-sized AuNPs (diameter: 60 nm) near FITC. However, when the single-stranded region was cleaved by the trans-cleavage effect of activated CRISPR/Cas12a due to target gene detection, the large-sized AuNPs were released, and the remaining nanocomplex comprised small-sized AuNPs (diameter: 20 nm). FITC exhibited strong fluorescence emission via the MEF effect from the small-sized AuNPs located 7 nm away from it. The AuNP-based biosensing system demonstrated remarkable biosensing properties for detecting BRCA-1 released from breast cancer cells (MCF-7). This was enabled by the use of the CRISPR/Cas12a-based fluorescent biosensor, which exhibited a high level of sensitivity that reached the femtomolar level. Moreover, a previous report revealed that Au nanobipyramids (AuNBPs) were employed in a CRISPR/Cas12a-based biosensor that utilized their MEF property to detect HPV16 in a colorimetric approach with high sensitivity [71]. Another study reported a simple and rapid CRISPR/Cas12a-based colorimetric biosensor that exhibits a colorimetric change in AuNP aggregation induced by the trans-cleavage effect of CRISPR/Cas12a upon target gene detection [72]. Also, in recent years, numerous SARS-CoV-2 biosensors have been studied using various nanomaterials [73, 74]. To detect the SARS-CoV-2 viral RNA using CRISPR/Cas and plasmonic nanomaterials, several studies were reported using Cas12a and Cas13a, respectively [75, 76]. In addition, by combination of AuNPs and magnetic nanomaterials, plasmonic AuNPs with magnetic property were utilized with CRISPR/Cas12a to detect viral DNA (HPV16) recently [77].

Although only a few studies have been reported to date, the recently developed CRISPR/Cas-based fluorescent/colorimetric nanobiosensors have exhibited excellent biosensing performance in detecting important biomarkers, including miRNAs. Since research on the application of plasmonic nanomaterials to CRISPR/Cas-based biosensors is relatively new, we expect to see more novel CRISPR/Cas-based nanobiosensors composed of plasmonic nanomaterials in future research.

## CRISPR/Cas-Based SERS and LSPR Nanobiosensors for Detecting Disease Biomarkers

**Fig. 5:**
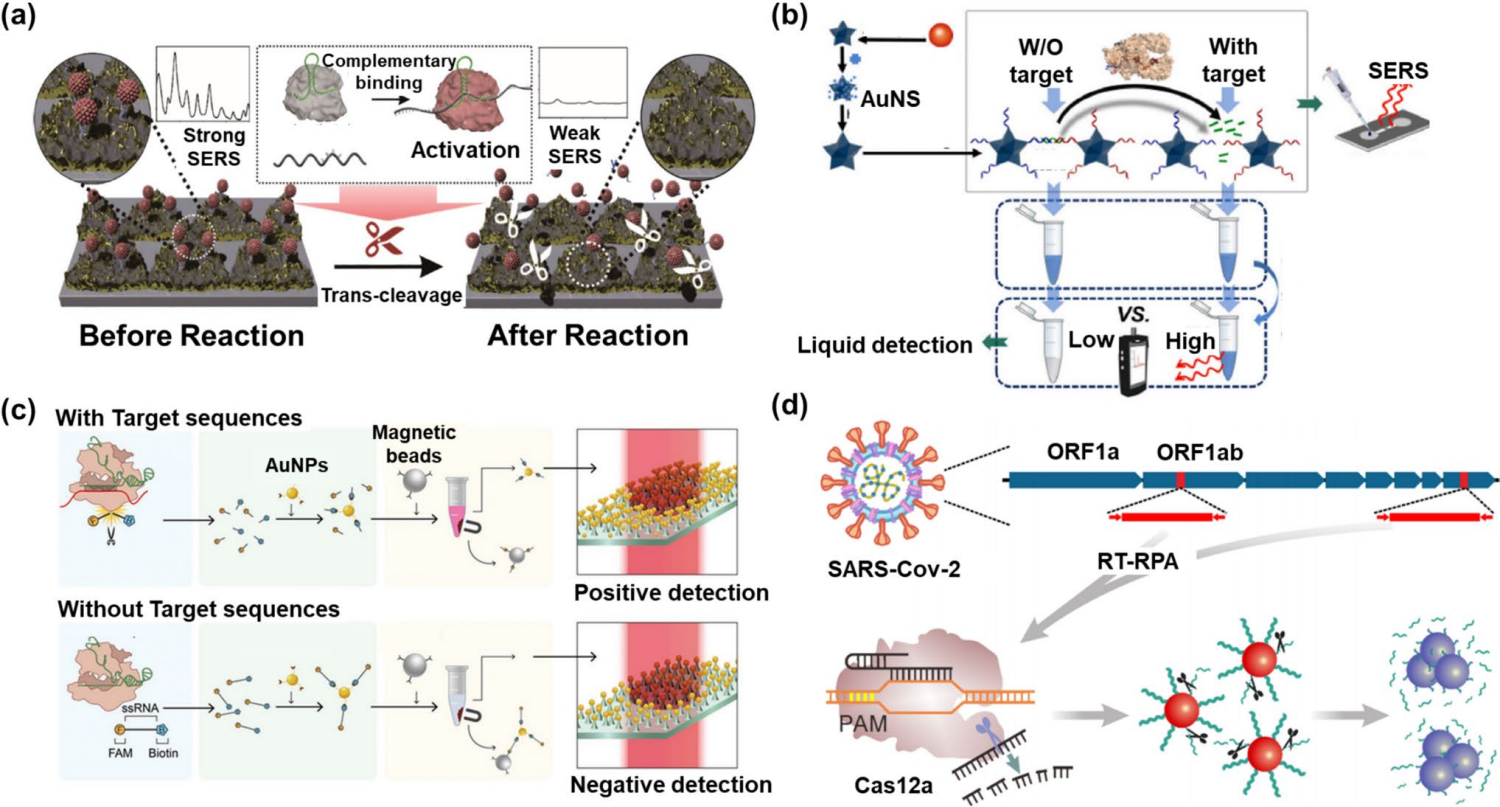
CRISPR/Cas-based SERS and LSPR nanobiosensors using plasmonic nanomaterials. **a** Schematic of the GO-Au nanoflower platform for CRISPR-Cas12a-assisted viral DNA detection using the SERS-based signal enhancement method. Reproduced with permission [80]. Copyright 2021, American Chemical Society. **b** Schematic illustration of the CRISPR/Cas-based SERS nanobiosensor with Au nanostars on an LFA platform. Reproduced with permission [84]. Copyright 2022, Elsevier. **c** Schematic of the LSPR-based biosensor that utilizes the interaction of magnetic particles and Au nanomushrooms. Reproduced with permission [85]. Copyright 2022, Wiley. **d** Schematic of the AuNP-assisted colorimetric biosensor based on CRISPR-Cas12a-based LSPR. Reproduced with permission [72]. Copyright 2021, American Chemical Society

In parallel with research on fluorescent biosensors, extremely sensitive SERS-based biosensors have been developed by incorporating nanomaterials, such as Au-coated magnetic NPs and Au core–satellite nanoclusters, into CRISPR/Cas-based biosensing systems [78, 79]. In one study, a SERS-active nanoarray and AuNPs were modified with highly accumulated Raman probes to develop an amplification-free CRISPR/Cas12a-based SERS biosensor for viral DNA detection [80]. In the study, the authors introduced the highly SERS-active triangular Au nanoflower array to enhance the Raman signal from the Raman probes via electromagnetic and chemical enhancement effects. In addition, the Raman probes were efficiently accumulated on the AuNP, which was immobilized on the SERS-active triangular Au nanoflower array via ssDNA (Fig. 5a). In the presence of target viral DNAs (hepatitis B virus, HPV16, and HPV18), the activated CRISPR/Cas12a cleaved the ssDNA. The release of the accumulated Raman probe-modified AuNP from the triangular Au nanoflower array resulted in a significant decrease in the Raman signals. This SERS biosensor exhibited excellent sensitivity at the attomolar level without requiring amplification steps. Pan et al. [81] presented a tactful biosensing strategy that utilizes a target-responsive Prussian blue nanolabel and a CRISPR/Cas12a effector for the specific and highly sensitive detection of target DNA. This biosensor achieves an ultralow LOD of 224 aM, enabling precise and reliable detection of the target DNA. It is suitable for applications such as milk authenticity detection. Yin et al. [79] developed a highly sensitive and selective nucleic acid biosensor using chimeric DNA/RNA hairpins (displacers) that can be disrupted by activated CRISPR-Cas12a to liberate excessive RNA. This results in the disintegration of the core–satellite nanocluster comprising AuNPs and a drastic decrease in SERS intensity as signal readouts. The introduction of a magnetic core to the large AuNPs enhanced the detection sensitivity, resulting in an ultralow LOD of 1 aM. The developed biosensor demonstrates promise for nucleic acid detection applications. Next, in a recent study, CRISPR/Cas12a was used to develop a SERS-based LFA biosensor to detect HIV-1 with ultrasensitivity (0.3 fM sensitivity without amplification) [82]. In another study, a split aptamer of 17β-estradiol, an endocrine-disrupting chemical, was co-introduced with CRISPR/Cas12a and Raman dye to develop an LFA-based 17β-estradiol biosensor using SERS [83]. Zhuang et al. [84] demonstrated that RPA-Cas12a-μPAD is a highly sensitive and rapid bacterial detection platform that integrates RPA with Cas12a trans-cleavage to enable supersensitive SERS detection (Fig. 5b). The platform offers a reliable and efficient method for accurately testing food samples, with a low LOD of 3–4 CFU/mL and a dynamic detection range of 1–108 CFU/mL. These research efforts are expected to contribute to the development of CRISPR/Cas biosensors that can broadly measure several disease-related biomarkers with simple and high sensitivity.

In addition to the SERS effect, localized SPR (LSPR) has been employed in CRISPR-based biosensing applications because of its ability to intensify light–matter interactions and generate unique intensity spectra with shifting peak resonance wavelengths. Plasmonic nanostructures with a sub-wavelength thickness and localized resonance allow for the sensitive characterization of biomolecular interactions, making them ideal for sensitive biosensing applications and more advantageous than traditional SPR devices. In a previous study, a novel LSPR system was developed by coupling Au nanomushrooms and AuNPs, and it demonstrated a significant plasmonic resonant shift. This innovative setup accomplishes a redshift of 31 nm when saturated with AuNPs (Fig. 5c) [85]. This LSPR chip offers a straightforward, specific, isothermal, and label-free detection approach, thereby opening up new avenues for advanced multiplexing and highly sensitive molecular diagnostic systems, particularly in the context of detecting SARS-CoV-2 RNA targets. In a separate study, a hybridization chain reaction (HCR)-powered CRISPR/Cas12a-based biosensor was introduced for the colorimetric detection of serum prostate-specific antigen (PSA) via the LSPR phenomenon, and it exhibited a low LOD of 0.1 ng/mL [86]. The integration of the enzymatic-free and isothermal amplification of the HCR, the specific recognition and trans-cleavage activity of CRISPR/Cas12a, and the distance-dependent optical characteristics of AuNPs into a single sensing system establishes a robust foundation for the development of biosensors based on nonenzymatic and isothermally amplified CRISPR/Cas systems for the detection of protein cancer biomarkers. Furthermore, Zhang et al. [72] devised an RT-RPA coupled with CRISPR-Cas12a colorimetric assay for detecting SARS-CoV-2. This method utilizes DNA-modified AuNPs to specifically target the ORF1ab and N regions of the SARS-CoV-2 genome (Fig. 5d). The process involves the hydrolysis of capped DNA from the AuNPs during trans-cleavage, resulting in an SPR change that can be detected with both UV–Vis spectroscopy and the naked eye. This method demonstrates exceptional sensitivity (detecting as low as one copy of the viral genome sequence per test) and heightened specificity, effectively mitigating the occurrence of false positive results from other members of the beta coronavirus family.

## Conclusion and Future Perspectives

Up to now, various functional nanomaterials and biomolecules like aptamers have been studied to develop of ultrasensitive biosensors [91–93]. Among the candidates for biosensor development, CRISPR/Cas systems have demonstrated tremendous potential for use in the development of biosensors. The incorporation of plasmonic nanomaterials into CRISPR/Cas systems aids in overcoming the limitations of these systems, amplifying the signals, and enhancing the sensitivity of biosensors to detect various disease biomarkers, such as nucleic acids, small molecules, and proteins. Recently, there has been an increase in the number of studies devoted to the development of CRISPR/Cas-based nanobiosensors that comprise plasmonic nanomaterials. The integration of CRISPR/Cas systems and plasmonic nanomaterials suggests a promising approach to revolutionize the molecular diagnostics and monitoring of diseases, including genetic abnormalities, viral infections, and cancers. This review highlights recent studies on CRISPR/Cas-based nanobiosensors composed of plasmonic nanomaterials and discusses the biosensing mechanisms of CRISPR/Cas systems, widely used plasmonic nanomaterials, and CRISPR/Cas combined with plasmonic nanomaterials for the detection of disease biomarkers (Table 2).

**Table 2:** CRISPR/Cas-based nanobiosensors for disease biomarker detection

Technique	Cas type	Plasmonic nanomaterial	Target	Detection range	Sensitivity	Reference
Fluorescent/colorimetric nanobiosensors	Cas12a	AuNPs	African swine fever virus	N/A	1000 copies per µL	[90]
	Cas12a	AuNPs	HPV16 and HPV18	N/A	3.1 aM	[48]
	Cas12a	AuNPs	DNA	0.5 pM–100 nM	0.134 pM	[68]
	Cas12a	MXene–Au	DON	1–500 ng/mL	0.64 ng/mL	[69]
	Cas12a	AuNPs	BRCA-1 gene	1 fM–100 pM	0.34 fM	[37]
	Cas12a	AuNBPs	HPV16	10–500 pM	1.0 pM	[71]
	Cas12a	AuNPs	SARS-CoV-2	N/A	4 copies/μL	[75]
	Cas13a	AuNPs	SARS-CoV-2	N/A	3 fM	[76]
	Cas12a	Au@Fe_3_O_4_	HPV16	256 nM–250 pM	0.25 nM	[77]
SERS/LSPR nanobiosensors	Cas12a	Au nanoflower-GO-AuNPs	HPV16 and HPV18	1 aM–100 nM	1 aM	[80]
	Cas12a	Au@Ag core–shell NPs	Cow DNA	1 fM–10 nM	224 aM	[81]
	Cas12a	AuNPs	Orf-cDNA	10 aM–1 nM	10 aM	[79]
	Cas12a	SiO_2_@Au	HIV-1	1 fM–10 nM	0.3 fM	[82]
	Cas12a	Au@BDT@Au	17β-estradiol	1 pM–10 nM	180 fM	[83]
	Cas12a	AuNS	*Salmonella Typhimurium (S. Typhi)*	1–10^8^ CFU/mL	3 CFU/mL	[84]
	Cas13a	Au nanomushrooms	SARS-CoV-2 RNA	100 aM–1 fM	100 aM	[85]
	Cas12a	AuNPs	PSA	0.2–40 ng/mL	0.1 ng/mL	[86]
	Cas13	AuNPs	SARS-CoV-2 genome	1 pM–1000 nM	1 pM	[72]
	Cas12a	AuNPs, ZnO Nanorod	miR-21	0.5 fM–100 pM	0.18 fM	[87]
	Cas13	AuNPs	SARS-CoV-2 RNA	10^3^–10^8^ copies/mL	518 copies/mL	[88]
	Cas12a	AuNPs	P53 DNA	1 fM–10 nM	0.40 fM	[89]

**Fig. 6:**
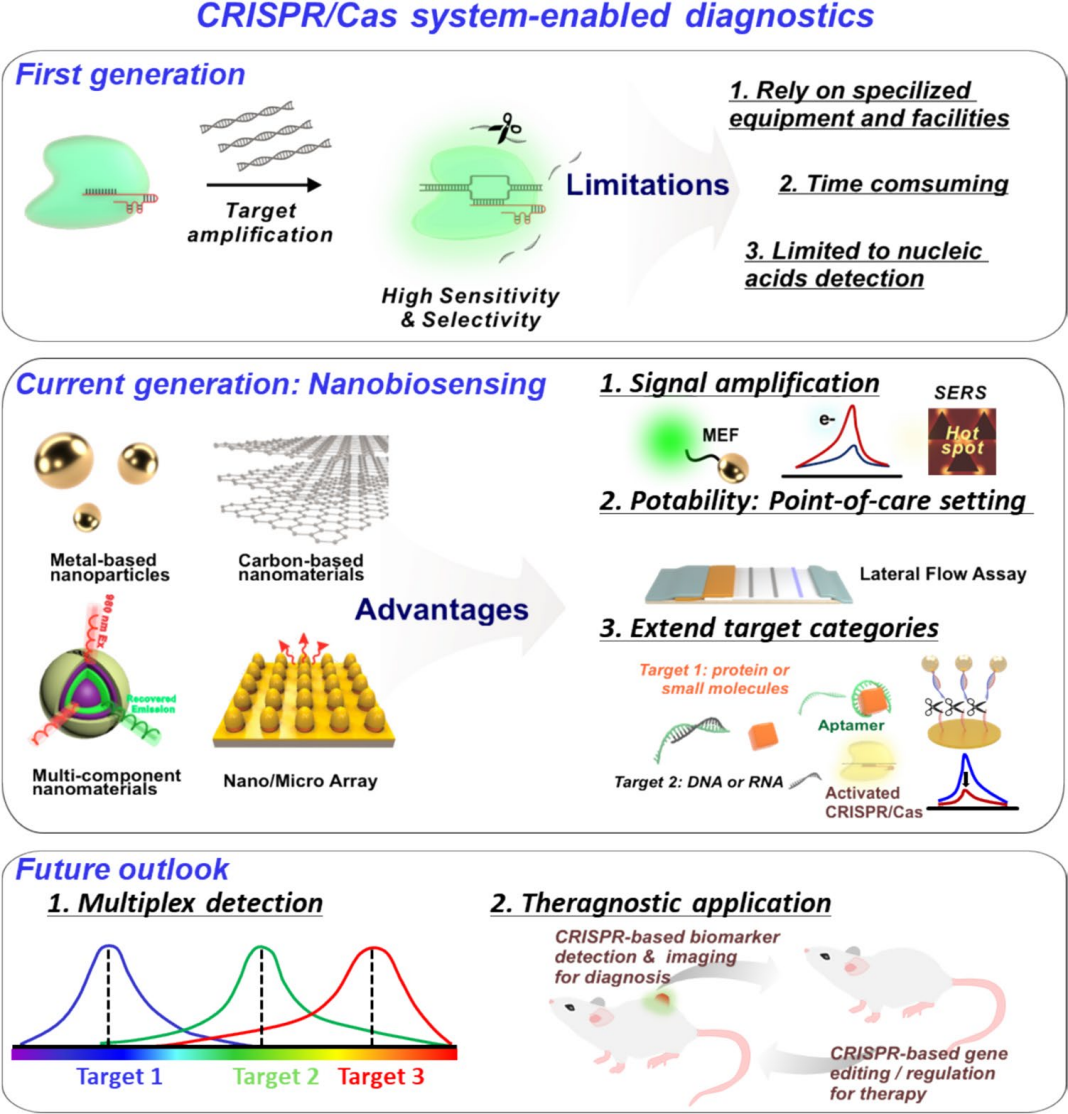
Overview of the current generation and the future outlook of CRISPR/Cas-based nanobiosensors that utilize plasmonic nanomaterials for advanced diagnostics and other applications

Although CRISPR/Cas-based nanobiosensors composed of plasmonic nanomaterials offer several advantages for disease biomarker detection, their precision and sensitivity still require improvement (Fig. 6). Most CRISPR/Cas-based biosensors incorporate nucleic acid amplification steps for sensitive target detection. Nucleic acid amplification-based assays, such as PCR, have been widely used for sensitive target detection, particularly in cell-based assays. However, their detection is not entirely precise, accurate, or sensitive, possibly because of significant limitations, which include the possibility of false positive results as well as time and cost inefficiencies caused by complicated procedures. The use of CRISPR/Cas systems can reduce the disadvantages associated with nucleic acid amplification; nonetheless, overcoming these limitations is challenging. Overcoming these challenges necessitates a careful evaluation of the specific requirements and the further development and introduction of novel nanomaterials other than plasmonic nanomaterials into CRISPR/Cas systems. These advancements could provide a comprehensive solution for eliminating nucleic acid amplification. Furthermore, beyond the techniques discussed in this review, other detection methodologies, such as electrochemistry, can be combined with CRISPR/Cas-based nanobiosensors that comprise innovative nanomaterials in order to overcome the limitations of the amplification stage. In light of all their distinct advantages, we anticipate that these nanobiosensors will ultimately replace current traditional molecular diagnostic platforms, such as PCR.

Further improvement in CRISPR/Cas nanobiosensors is required to enable multiplex detections of different disease biomarkers. Multiplex detection is a crucial aspect of measuring several biomarkers simultaneously, which is essential for efficient disease assessment. By harnessing its potential, this technology can significantly reduce the number of samples, analysis time, and human resources required to create sophisticated biosensors, allowing for more precise and sensitive diagnoses. The use of different nanomaterials that generate various signals makes it possible to measure multiple biomarkers simultaneously. For example, quantum dots can be tuned to display different fluorescence emission wavelengths, allowing biomarkers to be identified based on each emission wavelength. This enables the evaluation of multiple biomarkers simultaneously. Integrating this technique with a CRISPR/Cas-based biosensor would enable the simultaneous measurement of multiple biomarkers. It would also enable existing CRISPR/Cas-based biosensors to provide a sensitive and straightforward measurement. Additionally, the recombination technique of CRISPR/Cas systems can be used to achieve selective collateral cleavage of nucleic acids for the multiplex detection of biomarkers [9]. Integrating multiplex detection properties with advanced CRISPR/Cas-based biosensors could revolutionize the field of disease diagnosis. Moreover, CRISPR/Cas systems can be used for effective theragnostic applications when their biosensing functions are combined with their gene-editing function. They can provide an accurate diagnosis, create high-quality imaging, and deliver effective gene therapy, but this is yet to be explored. In addition to the detection of disease biomarkers, they also have the potential to be applied in the biomedical field such as the cell culture monitoring [94]. These features can also enhance the utility of CRISPR/Cas systems in biosensing, therapeutic and biomedical applications. We expect that a nanomaterial-integrated CRISPR/Cas biosensing system could become a prominent market-leading technology in the fields of molecular diagnosis and biomedicine through research into CRISPR/Cas systems leveraging the inherent and diverse properties of nanomaterials, including versatile plasmonic nanomaterials.

## Data Availability

Not applicable.
